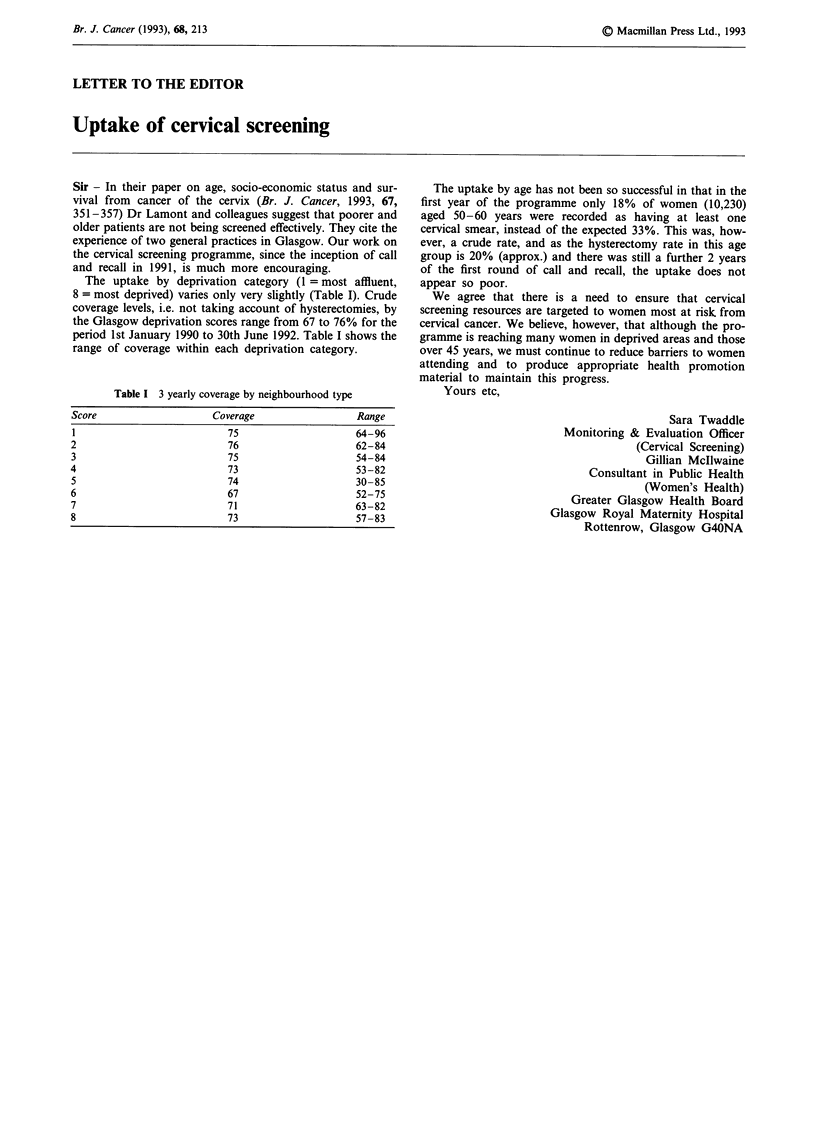# Uptake of cervical screening.

**DOI:** 10.1038/bjc.1993.316

**Published:** 1993-07

**Authors:** S. Twaddle, G. McIlwaine


 © Macmillan Press Ltd., 1993

LETTER TO THE EDITOR

### Uptake of cervical screening

**Sir** – In their paper on age, socio-economic status and survival from cancer of the cervix (*Br. J. Cancer*, 1993, **67**, 351–357) Dr Lamont and colleagues suggest that poorer and older patients are not being screened effectively. They cite the experience of two general practices in Glasgow. Our work on the cervical screening programme, since the inception of call and recall in 1991, is much more encouraging.

The uptake by deprivation category (1 = most affluent, 8 = most deprived) varies only very slightly (Table I). Crude coverage levels, i.e. not taking account of hysterectomies, by the Glasgow deprivation scores range from 67 to 76% for the period 1st January 1990 to 30th June 1992. Table I shows the range of coverage within each deprivation category.

**Table I** 3 yearly coverage by neighbourhood type

| *Score* | *Coverage* | *Range* |
|---|---|---|
| 1 | 75 | 64–96 |
| 2 | 76 | 62–84 |
| 3 | 75 | 54–84 |
| 4 | 73 | 53–82 |
| 5 | 74 | 30–85 |
| 6 | 67 | 52–75 |
| 7 | 71 | 63–82 |
| 8 | 73 | 57–83 |

The uptake by age has not been so successful in that in the first year of the programme only 18% of women (10,230) aged 50–60 years were recorded as having at least one cervical smear, instead of the expected 33%. This was, however, a crude rate, and as the hysterectomy rate in this age group is 20% (approx.) and there was still a further 2 years of the first round of call and recall, the uptake does not appear so poor.

We agree that there is a need to ensure that cervical screening resources are targeted to women most at risk from cervical cancer. We believe, however, that although the programme is reaching many women in deprived areas and those over 45 years, we must continue to reduce barriers to women attending and to produce appropriate health promotion material to maintain this progress.

Yours etc,

Sara Twaddle
Monitoring & Evaluation Officer
(Cervical Screening)
Gillian McIlwaine
Consultant in Public Health
(Women's Health)
Greater Glasgow Health Board
Glasgow Royal Maternity Hospital
Rottenrow, Glasgow G40NA